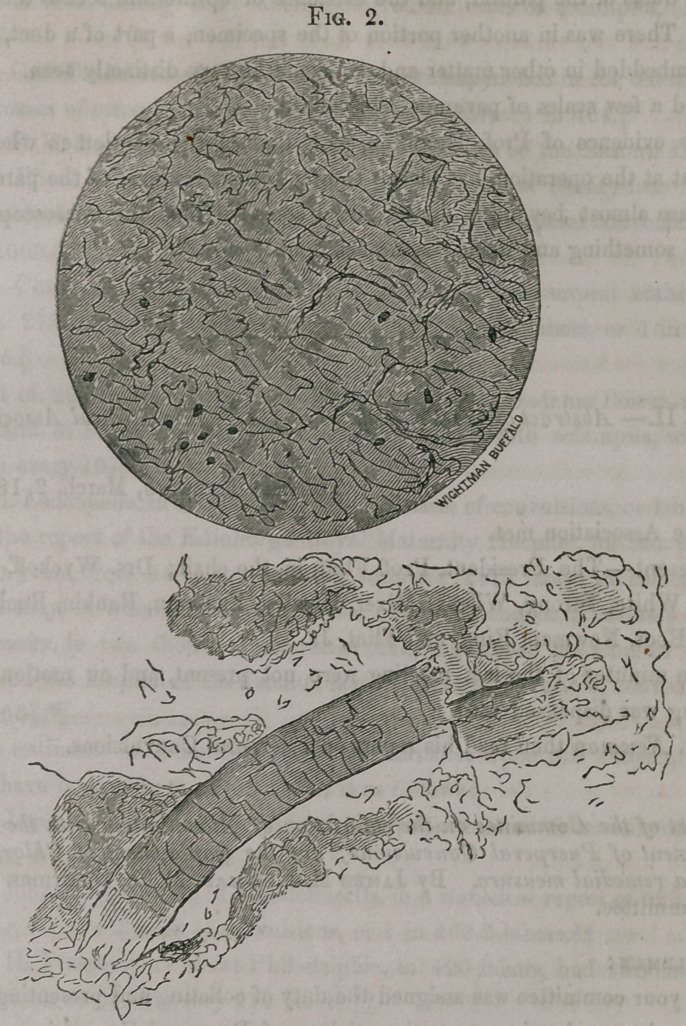# Tumor of the Parotid Gland

**Published:** 1858-05

**Authors:** Frank H. Hamilton


### BUFFALO MEDICAL JOURNAL

AND

### MONTHLY REVIEW.

**VOL. 13.** MAY, 1858. **NO. 12.**

### ORIGINAL COMMUNICATIONS.

ART. I.—*Tumor of the Parotid Gland. Complete Extirpation of the Mass; including the entire Gland.* By FRANK H. HAMILTON, M. D.

R. A. P., æt. 28, of Toronto, C. W., was struck by a small stone thrown by a lad, on the right side of the neck, in the year 1832. The blow was received at a point about one-quarter of an inch below the lobe of the ear, and directly over the seat of the parotid gland. The injury was trivial, and the slight wound soon healed. About one year from this, Mr. P. discovered a small tumor in the situation of the scar. This has continued to grow more or less rapidly from that time until it was removed. It was never painful until very lately.

Five months ago it had attained the size of a goose egg, and was situated mainly in front of the ear and upon the cheek. Stimulating poultices were then applied, which were always very painful, and after they had been continued one month a tumor was found to be forming back of the ear. The original tumor was now scarified at several points, and to the depth of half an inch; and various caustics, such as nitrate of silver, sulphate of copper, etc., were thrust into the wounds. The poultices were also continued. Nearly at the same time, other masses were seen rapidly forming below, and under the chin, and a complete paralysis of the right side of the face with partial loss of voice, accompanied with difficulty of deglutition ensued.

This was his condition when in Feb., 1858, he applied to Dr. Hamilton for relief. He was then quite feeble; deaf in his right ear, and with occasional bloody discharges both from his ear and mouth. The tumor, or the successive masses, extended from a point on the face above the ear, to near the cricoid cartilage, and its breadth under the ear was nearly equal to its length, occupying the upper third of the neck on the right side. It covered also nearly one-third of the face.

Hoping that the recent formations were merely inflammatory exudations, Mr. P. was sent home for a few day and submitted to treatment, in the hope that they might be reduced.

Feb. 9, 1858, he returned to Buffalo, with only a slight change in the condition of the tumor.

*Operation*, in presence of Drs. Wilcox, Flint, Jr., and Bartlett. Mr. P. preferred not to take any anæsthetic.

Five crucial incisions were made across the two longest diameters of the tumor and the masses carefully brought to view. It was soon ascertained that the sterno cleido mastoid must be severed completely in order to enucleate the lower tumor. A large portion of the upper tumor lay beneath the digastricus, and it became necessary to sever it also. The branches of the external carotid were tied from below as they were brought into view. In all about twenty ligatures were applied. The loss of blood did not exceed half a pint, owing to the care taken to secure the vessels as soon as they were seen.

When the whole was removed the space originally occupied by the parotid gland was entirely empty; the bones, cartilages, ligaments of the joints, &c., constituting the walls of this cavity being fully exposed. The common sheath of the carotid artery and jugular vein were laid bare down to nearly the middle of the neck.

From the body of the tumor, extending forwards beneath the ascending ramus of the lower jaw, fairly into the buccal cavity, was a prolongation of the same structure which Dr. Hamilton tore out with his finger.

The portio dura of the seventh pair was brought completely into view during the dissection, lying in the body of the upper tumor; and after some hesitation it was cut in two.

The wound was closed with sutures, and dressed with simple cerate, &c.

On the fourth day erysipelas began to develop itself around the wound, which extended finally over the whole face and head. On the eleventh day it had nearly disappeared, and the cavity was closing finely.

His power of speech and of deglutition is now nearly recovered, but the paralysis of the right side of the face and of the right eyelids still continues. He is able to ride out and his recovery is beyond a reasonable doubt.

*Examination of the Product.* Large portions, especially of the recent formations, were of a solid consistence, but granular and easily broken with the finger, appearing like fibro-plastic tissue, or simple inflammatory exudation: so also the greater portion of the original tumor was solid, or only slightly softened, but other portions were much more softened.

*Dr. Hamilton* remarked that he did not intend here to discuss the possibility of this operation, although it was still doubted by Erichsen, and, perhaps by others. Velpeau had collected thirty-five cases in Europe in which it was believed that the parotid had been removed; and in this country it is claimed to have been removed thirty-eight times. Among the operators are the names of surgeons whose authority and opinions we have been accustomed to respect, and we cannot doubt but that they have done what they claim to have done: such are the names of Mott, Homer, Randolph, N. R. Smith, each of whom have operated once successfully, and of George McLellan, who operated eleven times.

*Dr. Wilcox* said that he was present at the operation, and that he saw the portio dura passing through the substance of the mass, and that he had no doubt the parotid gland was removed. Nothing remained behind the jaw after its removal, but the walls of the cavity with nerves, vessels, &c. There was no gland left; his finger penetrated to the mouth.

*Prof. White* said that he had examined the mass removed, and he had no doubt but that it was the parotid gland.

### Microscopic Appearances. By Austin Flint, Jr., M. D.

Prof. Hamilton kindly presented me with a portion of this tumor, cut from the spot which might have been the parotid gland, hardened and changed, of course in its character, by the progress of the disease. This specimen, however, was unfortunately laid in a warm, dry place, and before I had an opportunity of examining it microscopically, it was almost entirely desiccated.

The readers of this Journal will recollect, however, in the proceedings of the Buffalo Medical Association, an account of the microscopic appearances in some specimens of uterine mucous membrane sent to Prof. White by Dr. Congdon. These specimens had become entirely dried, but in being subjected to the action of a fluid resembling the liquor sanguinis, for a few hours; entirely resumed their original gross and microscopical appearances, so as to present a beautiful demonstration of the anatomical characteristics of mucous membrane. I am inclined to think that nearly all morbid specimens can in a like manner be brought back to their original state, after having been dried, by the action of a slightly saline fluid.

This specimen was treated in the same way as those sent by Dr. Congdon, *i. e.*, it was placed in a slightly saline solution and allowed to remain for twenty-four hours. At the expiration of this time, it had recovered, to all appearances, its original state, and it was subjected to microscopic examination.

On making a section, and slightly pressing it, there exuded a juice or "*suc*" as it is called by the French; this was formerly supposed to be one of the important signs of cancer, but later observations have shown that it exists in many benign tumors. A portion of this juice was then placed between two plates of glass and submitted to the microscope, a magnifying power of 282 diameters, being used. There appeared in the field a quantity of amorphous granules or organic molecules, having the Brunonian movement, a few exudation corpuscles, conglomerate corpuscles, pus corpuscles, with a number of fibro-plastic cells and free fibro-plastic nuclei. (See fig. 1.)

A small portion was then taken from the substance of the tumor and torn up with dissecting needles, (the tearing communicated to the fingers something of the sensation of a fibrous structure.) This portion having been carefully separated, moistened and covered with a thin plate of glass, was submitted to microscopic examination, the magnifying power being now 581 diameters.

In one part of the field, when the structure had not been very thoroughly torn up by the needles, a fibrous structure was apparent, enclosing spaces which were occupied by fibro-plastic cells, nuclei, &c. The enclosing structure consisted chiefly of the ordinary white inelastic fibre, or the element of areolar tissue. On removing the field to a portion of the specimen which had been more effectually torn up, and where the anatomical elements were not so crowded, there could be seen fibro-plastic cells, free nuclei, a few pus and exudation corpuscles, and organic molecules, having the Brunonian movement. I have attempted to represent some of the fibro-plastic cells and free nuclei in Fig. 1.

FIG. 1.

FIG. 1. Magnifying power, 571 diame.ers.

*A.* Free fibro-plastic nuclei.

*B.* Fusiform fibro-plastic cells.

The cells which are not lettered are probably epithelium, which has assumed some of the eccentric shapes which it sometimes does.

I would remark that the decided appearance of fibrous structure did not exist in all portions of the specimen, but that I saw it in a single place only: the pus corpuscles and inflammatory corpuscles were also more numerous in this portion. In other portions of the specimen the fibro-plastic cells and free nuclei were by far the most abundant.

The microscopic appearances of this specimen would, by a great many, be mistaken for cancer; some going so far as to regard every caudate cell as cancerous. Lebert, however, pointed out the *fibro-plastic* element, which is natural to healthy tissue, but in some forms of disease becomes increased in quantity, as in the present case. This is called one of the accessory anatomical elements, and it has now become a principal element, by a morbid process.

Robin describes three varieties of fibro-plastic elements:

1st. The free fibro-plastic nuclei;

2d. The fusiform fibro-plastic cells; and

3d. The fibro-plastic cellules.

The first two varieties we always find; but the last is more infrequent, though we often find it, and then always the other varieties in connection with it. The difference between the fibro-plastic cell and the true cancer cell is very marked, by an actual comparison; but it must be acknowledged, that from ordinary book descriptions, it would be almost impossible to distinguish between them. One of the most distinct points of difference is the size; the cancer cell is a great deal larger than the fibro-plastic: its nucleus is also proportionally larger, and it frequently has two or three immense nucleoli: their shape, however, is very much the same.

From the examination which I have made of this tumor, the microscopic appearances being confirmed by Prof. Flint, I have no hesitation in pronouncing it a fibro-plastic growth: as indeed its gross appearance led me to suspect. As such, though not malignant, it is liable to return, though by no means with the nearly absolute certainty with which true cancerous formations are reproduced. The white inelastic element is, I believe, frequently, if not always, found in some quantity in such formations. The exudative corpuscles and pus corpuscles, which existed in small quantities in some portions, would seem to indicate that there was, or had been, inflammation in that portion of the structure. The pus nuclei, and the fibro-plastic cells were the characteristic anatomical feature. The organic molecules are met with in nearly all tumors.

A day or two after the first examination, in looking at a portion again through the microscope, which presented nearly the same appearances which I have just described, I discovered evidences of glandular structure. The appearances are represented in Fig. 2.

The lower figure was drawn with a magnifying power of 60 diameters, and represents a glandular duct, which in all probability belonged to the parotid. The tube measures 1–125 of an inch or a little less than 1–10 of a line in diameter. It is surrounded by amorphus granular matter and is broken at one part.

The upper figure represents a portion of the tube subjected to a magnifying power of 571 diameters. In this, we see in the greater part of the field, a confused mass, but by close examination we are able to make out, in some places, the boundaries and the nuclei of the pavement epithelium. I have attempted to represent the actual appearances as accurately as possible, in these figures, taking no pains to make the appearances more distinct than they actually were. All these observations were made in the presence of, and confirmed by, Prof. Flint.

Fig. 2.

The exceeding difficulty in removing the parotid gland, and the fact that its possibility has been denied by some of our most eminent surgeons, render exceedingly important every iota of testimony which can be brought to bear upon this subject.

The microscopic testimony in this case, seem to me to be exceedingly important. The existence of glandular structure in that situation, being proof that some of the gland had been removed; and as after the operation there was no parotid, it is undoubtedly the fact that all the gland was removed. The size of the duct corresponds with the measurements given by Kölliker of the ducts of the parotid, and the existence of epithelium settles the question. There was in another portion of the specimen, a part of a duct, but it was embedded in other matter and could not be very distinctly seen. I also noticed a few scales of pavement epithelium.

The evidence of Prof. Hamilton, with that of the gentlemen who were present at the operation, has placed the fact of the removal of the parotid in this case almost beyond a doubt, but I conceive that the microscope has added something and made "assurance doubly sure."

## Figures and Tables

**Fig. 1. f1:**
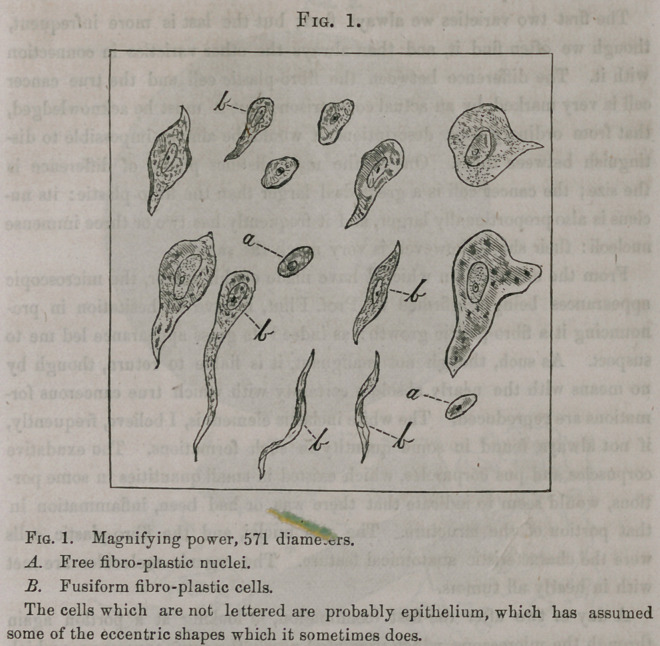


**Fig. 2. f2:**